# Clinical Progress in Virotherapy: Application and Future Prospects in Head and Neck Cancer

**DOI:** 10.3390/ijms27156682

**Published:** 2026-07-27

**Authors:** Yoshiaki Yura, Masakazu Hamada

**Affiliations:** Department of Oral & Maxillofacial Oncology and Surgery, Graduate School of Dentistry, The University of Osaka, Suita 565-0871, Osaka, Japan; hamada.masakazu.dent@osaka-u.ac.jp

**Keywords:** head and neck cancer, virotherapy, oncolytic virus, non-replicating virus, cancer vaccine, herpes simplex virus, adenoviruses, vaccinia virus, clinical trials

## Abstract

Virus-based cancer therapy (virotherapy) is currently being researched as a novel form of immunotherapy and has already entered the clinical application phase. Among various types of virotherapy, oncolytic virotherapy involves infecting tumors with tumor-selective viruses, such as herpes simplex virus, adenoviruses, and vaccinia virus, and utilizing their replicative capacity to induce cell destruction within tumors. In addition, this therapy aims to enhance tumor immunity by changing the tumor microenvironment through viral infection. Genetic deletion in viruses is used to reduce their virulence and confer tumor selectivity, while the expression of foreign genes is utilized to enhance antitumor effects. Oncolytic viruses for head and neck cancer (HNC) are administered locally or systemically and are sometimes used as adjuvant therapy or in combination with immune checkpoint inhibitors. Another form of virotherapy involves non-replicating viruses, which are used to produce antitumor cytokines or as cancer vaccines expressing tumor antigens. Research on the efficacy of cancer vaccines in preventing postoperative recurrence is currently underway. A number of challenges have yet to be overcome for further advances in virotherapy, including the efficient delivery of viruses to tumor cells, avoiding viral inactivation in the bloodstream, ensuring efficient replication of the virus, and enhancing antitumor immunity. The development of effective strategies based on the findings of clinical studies will lead to improvements in virotherapy for HNC.

## 1. Introduction

Head and neck cancer (HNC) refers to malignant tumors that develop in areas including the oral cavity, pharynx, larynx, nasal cavity, maxillary sinuses, and salivary glands. It ranks as the sixth most common cancer worldwide [1]. The majority of cases are squamous cell carcinomas (SCC), accounting for 90% of all cases; however, the category also includes malignant tumors, such as salivary gland cancer, mucosal melanoma, sarcomas, and malignant lymphomas [2]. Causes of the disease include tobacco use, alcohol consumption, Epstein–Barr virus (EBV), and human papillomavirus (HPV). Treatment typically involves surgery, radiotherapy, and chemotherapy, although the specific approach varies depending on the site of origin and the disease stage. After tumor resection, if risk factors, such as close margins, multiple lymph node metastases, vascular invasion, and nerve invasion, are present, chemoradiotherapy is added as an adjuvant therapy. However, in a follow-up study with a median duration of 45.9 months, the estimated incidence of local or regional recurrence was reportedly 31% with radiotherapy alone and 18% with cisplatin and radiotherapy, indicating that the risk of recurrence persists even after adjuvant therapy [3]. The re-irradiation of recurrent lesions is difficult, and chemotherapy becomes the primary treatment, similar to the management of distant metastases.

In recent years, the efficacy of immune checkpoint inhibitors (ICIs), such as anti-PD-1 and anti-PD-L1 antibodies for cisplatin-resistant HNC, has been confirmed, and the treatment has received FDA approval. The ICI antibodies, nivolumab and pembrolizumab, bind to PD-1 expressed on cytotoxic T cells, thereby blocking the PD-1/PD-L1 pathway and restoring the body’s natural antitumor immune response against tumor antigens [4,5]. Although ICIs have been introduced into the treatment of advanced HNC, their efficacy remains limited, with response rates of approximately 23% [5]. However, systemic therapy outcomes have improved when ICIs are combined with chemotherapy. Consequently, ICI-based combination strategies, including molecular targeted agents and other immunotherapies such as oncolytic virotherapy, are now being actively investigated [6,7].

The mainstay of virus-based cancer therapy (virotherapy) is oncolytic virotherapy using replicating viruses. Clinical research is progressing in cases of refractory, advanced disease with no other treatment options, particularly melanoma, glioblastoma, pancreatic cancer, liver cancer, and colorectal cancer, which affect a large number of patients [8,9,10,11,12]. The replication of an oncolytic virus in tumor cells has been shown to induce immunogenic cell death, leading to the release of pathogen-associated molecular patterns, damage-associated molecular patterns, such as ATP and calreticulin, and tumor antigens into the tumor microenvironment (TME). Consequently, innate and adaptive immune cells converge in the TME, are activated, and transform the TME from a “cold” to a “hot” phase [13,14]. In HNC, clinical-level research is advancing with DNA viruses, such as herpes simplex virus (HSV), adenoviruses, and vaccinia virus, as well as RNA viruses including reovirus, Newcastle disease virus, and vesicular stomatitis virus [15,16,17]. Oncolytic virotherapy for HNC has primarily been used to treat advanced, recurrent, or metastatic cases. Although many clinical trials have already been registered, only a fraction of the findings obtained have been reported. Another approach to virotherapy involves the use of non-replicating viruses for inducing cytokine production at the tumor site and developing cancer vaccines. Cancer vaccines are targeting antigens, such as tumor-associated antigens (TAAs) common to cancers or tumor-specific antigens (TSAs), and are administered in the form of peptides, mRNA, or DNA to enhance antitumor immunity and eliminate tumor cells.

Comprehensive reviews of oncolytic virotherapy have been published [8,9,10,18,19]. Reviews on HNC are also available [16,17,20]. In this review, we focus on three types of DNA viruses: HSV, adenoviruses, and vaccinia virus, for which research as therapeutic viruses is progressing and also on research undertaken with both oncolytic viruses (replicating viruses) and non-replicating viruses. We will discuss the genetic modification of viruses used in virotherapy, their clinical outcomes in HNC and other tumor types, preclinical studies, virus-based cancer vaccines, and future prospects to address challenges identified in previous research.

## 2. Basic Properties of Parental Viruses Used in Virutherapy

### 2.1. HSV

HSV often manifests in the oral mucosa during primary infection, causing herpetic stomatitis. Following acute infection, the virus travels retrograde along nerve fibers to ganglia, where it establishes a latent infection and becomes the source of recurrent herpes. Of the two human HSV, HSV type 1 (HSV-1) and HSV type 2 (HSV-2), HSV-1 infects the oral cavity and other sites from early childhood, and most individuals possess antibodies against it; however, HSV-2, which is considered the cause of genital herpes, infects at a later age, and antibody prevalence among adults is low [21]. It consists of double-stranded DNA and possesses a large genome estimated to be at least 150 kb. Therefore, it may incorporate foreign genes of 30–40 kb. Major viral glycoproteins responsible for viral entry are gB, gC, gD, gH, and gL, which bind to various cellular receptors, including herpes virus entry mediator, nectin-1, nectin-2, and heparan sulfate. Infection occurs through cell adhesion and uptake of the virus. The virus may infect a wide range of cell types. Replication takes place within the nucleus (Table 1).

### 2.2. Adenoviruses

Adenoviruses are a group of viruses that cause upper respiratory tract infections. They contain a double-stranded DNA genome of 36 kb, with a diameter of 90–100 nm, and lack an envelope. There are seven species (groups), designated A through G. They are further divided into 114 types [22]. Viral DNA replication occurs in the nucleus (Table 1). The primary receptor is the coxsackievirus and adenovirus receptor (CAR); however, the virus also utilizes CD46, desmoglein 2 (DSG2), glycan GD1a, and polysialic acid as receptors. The receptor for adenoviruses in group C is CAR, while those in group B and some in group D bind to CD46. When administered into the bloodstream, adenoviruses have the ability to bind to soluble factors IX and X. While group C type 5 has primarily been used in virotherapy, group B serotypes, such as types 3 and 11, have come to be used in recent years [23,24]. Adenovirus early region 1A (E1A) and early region 1B (E1B) are early-expressed genes that play a key role in viral replication and subsequent gene transcription. The conserved region of the E1A protein binds to the cellular Rb protein, causing the Rb-E2F complex to dissociate. This activates the transcription of target genes by E2F, forcing the cell to progress into the S phase. On the other hand, the E1B-55 kDa protein encoded by E1B binds to and inactivates p53. The E1B-19 kDa protein has a similar function to Bcl-2 and inhibits apoptosis induced by adenoviruses. E3 prevents the elimination of virus-infected cells by the immune response. E4 encodes numerous proteins but is critical for the stability of viral RNA during the late stages of infection [22,24]. Since adenoviruses drive the cell cycle forward, viral replication may occur not only in dividing cells but also in cells in the quiescent phase.

### 2.3. Vaccinia Virus

Vaccinia virus has been used for many years as a vaccine against smallpox. It measures 270 × 350 nm and has a brick-shaped morphology. As a double-stranded DNA virus with a genetic size of 190 kb, it may incorporate foreign genes ranging from 25 to 40 kb. Unlike other DNA viruses, it replicates in the cytoplasm (Table 1). When the virus replicates within a cell and produces progeny viruses, particles of different morphologies, an intracellular mature virion (IMV), intracellular enveloped virion, and extracellular enveloped virion, are formed through multiple maturation stages. Vaccinia virus, used as an oncolytic virus, consists predominantly of IMVs, which are highly stable even during the purification process. Since the virus adsorbs and enters cells via multiple cell surface molecules, it has affinity for a wide range of cells. The corresponding receptors differ depending on the morphology of the viral particle. Viral H3L and A27L bind heparin sulfate, and D8L binds to chondroitin sulfates. Vaccinia virus replicates rapidly and lyses infected cells. It may be administered intravenously to cancer patients without antibodies [25,26,27]. To enhance safety, thymidine kinase (TK) and ribonucleotide reductase (RR) have been deleted from the viral genome. The viral growth factor VGF is an EGF homolog that activates the EGFR-RAS pathway and promotes cell proliferation. Even when VGF is deleted, the virus retains its oncolytic activity against tumor cells, while its replication and toxicity are reduced in normal cells [26,27].

## 3. Current Status of Clinical Applications of Oncolytic Virotherapy and Preclinical Studies of Emerging Oncolytic Viruses

### 3.1. Clinical Study Using Replicating Oncolytic Viruses for HNC and Their Virological Characteristics

#### 3.1.1. Clinical Study Using Replicating HSV for HNC

Many oncolytic HSV-1s used clinically lack the neurotoxic gene RL1 encoding ICP34.5 [28]. A number of studies have been conducted to elucidate the mechanisms underlying the tumor selectivity of ICP34.5-deleted HSV-1. When HSV-1 infects a cell, double-stranded RNA-dependent protein kinase (PKR) is activated, phosphorylating eukaryotic initiation factor 2 alpha (eIF2α) and halting the translation of viral and host cell proteins. However, during this process, ICP34.5 recruits protein phosphatase-1 to dephosphorylate phosphorylated eIF2α and allows protein synthesis and, thus, viral replication to proceed. If a virus lacking ICP34.5 infects normal cells, protein translation is halted and viral replication is prevented. However, since the Ras/Raf/MEK/ERK pathway, which inhibits PKR activation, is activated in tumor cells, viral replication occurs [29]. Growth arrest and DNA damage-inducible protein 34 (GADD34) is induced by the endoplasmic reticulum (ER) stress response and functions to dephosphorylate phosphorylated eIF2α. The ER stress response is strongly active in tumor cells; therefore, GADD34 compensates for the ICP34.5 deficiency, allowing the replication of ICP34.5-deficient viruses [30]. ICP34.5 also binds to Beclin-1 to inhibit autophagy. In normal cells, ICP34.5-deficient viruses are degraded by activated autophagy; however, in tumor cells where autophagy regulation is disrupted, viruses are not degraded and replicate [31]. Furthermore, ICP34.5 associates with and inactivates stimulator of interferon genes (STING), which leads to the down-regulation of interferon regulatory factor 3 and IFN responses. Since tumor cells have a weak IFN response, they may support viral replication even when ICP34.5 is deficient [32]. An oncolytic HSV-1 talimogene laherparepvec (T-vec) was used in clinical trials for melanoma. In addition to lacking two copies of ICP34.5, it lacks the US12 gene encoding ICP47, a protein that inhibits antigen presentation. Consequently, the adjacent US11 gene is expressed as an immediate-early gene, thereby enhancing viral replication. Furthermore, granulocyte-macrophage colony-stimulating factor (GM-CSF), regulated by a CMV promoter, is integrated into the ICP34.5 deletion site; when produced in infected cells, it acts to recruit immune cells [33]. Since the efficacy of the local administration of T-vec on unresectable advanced melanoma was demonstrated in clinical trials, T-vec was approved in the United States in 2015.

The findings of a clinical study investigating the effects of T-vec on HNC were reported in 2010 (Table 2). This phase 1/2clinical trial was conducted on patients newly diagnosed with stage III/IV head and neck SCC. In addition to standard surgical resection and subsequent chemoradiotherapy including cisplatin, 17 patients received four preoperative doses of virus administration to metastatic cervical lymph nodes. The virus was not administered to the primary mucosal tumor. The viral dose varied by cohort, ranging from 10^6^ to 10^8^ plaque-forming units (PFU). Local control was achieved, and at 29 months, disease-specific survival was reported in 82.4% of patients. The administration of T-vec to the cervical lymph nodes was found to be a potentially effective neoadjuvant therapy [34]. Subsequently, the same group treated 36 patients with chemotherapy-resistant recurrent or metastatic HNC using T-vec in combination with the ICI pembrolizumab. T-vec was administered into tumors. A confirmed partial response (PR) was observed in 13.9%; however, 27.8% died early, making it impossible to evaluate efficacy. No added benefit was observed over ICI monotherapy alone [35]. These studies demonstrate that patient selection for the T-vec treatment significantly affects its efficacy.

Based on the T-vec structure, many oncolytic HSV-1s expressing pro-inflammatory cytokines, such as IL-2, IL-12, and IL-15, have subsequently been developed. SKV-012 lacks ICP34.5 and ICP47 and has a structure in which ICP34.5 is regulated by the survivin core promoter in addition to IL-12 under the CMV promoter, thereby selectively enhancing viral infection in tumors expressing survivin. A phase 1 clinical study was conducted on patients with advanced solid tumors, including HNC, with the virus administered intratumorally at concentrations of 10^6^ or 10^8^ PFU/mL. Among the six patients, three achieved PR, one had stable disease (SD), and two showed progressive disease (PD). Increases in CD8^+^ T cells, dendritic cells (DCs), and PD-L1 were observed in the TME [36]. VT1093 is an oncolytic HSV-1 that lacks ICP34.5 and ICP47 and incorporates the anti-PD-1 antibody gene. Upon viral infection, it produces anti-PD-1 antibodies in the TME to locally reverse immunosuppression, thereby avoiding the adverse events (AEs) associated with systemic ICIs. In a phase 1 trial targeting recurrent HNC, 1 × 10^7^, 1 × 10^8^, and 5 × 10^8^ PFU were administered intratumorally. Single and multiple doses were both administered, and although the assessment was based on a short 28-day monitoring period, there were four cases of SD and one of PD, resulting in a disease control rate of 57.1% (4/7). The most common AEs were fever and fatigue, with no grade 3 or higher events [37]. HF-10, an attenuated oncolytic HSV-1 lacking UL43, UL49.5, UL55, UL56, and latency associated transcript, was administered intratumorally to 16 patients with advanced disease, including HNC, at the doses of 10^4^ to 10^6^ PFU. No serious AEs were observed. Among the eight pancreatic cancer cases, there was one PR, three SD, and four PD [38]. T3011 is a multifunctional, genetically modified oncolytic virus based on HSV-1. To enhance tumor selectivity, it lacks one copy of ICP34.5, retains ICP47, and incorporates the IL-12 and PD-1 antibody genes. It is designed to produce IL-12 within the tumor to activate NK cells and CTLs, and to produce PD-1 antibodies within infected tumor cells to induce localized immune checkpoint inhibition. A phase 1/2a clinical trial was conducted to evaluate the virus as a monotherapy administered directly into the tumor in patients with advanced solid tumors, including recurrent or metastatic HNC. In the Part III group, the virus was administered at a dose of 1 × 10^8^ PFU/mL. The objective response rate (ORR) was 6.8%, the disease control rate was 39.2%, and the median duration of response was 10.6 months; no serious adverse events were reported. Compared to conventional treatments, favorable results were observed in advanced cases [39].

In addition to HSV-1, HSV-2 was also used at the clinical level. OH2 is a construct that lacks HSV-2 ICP34.5 and ICP47 and incorporates GM-CSF. A phase 1/2study was conducted on patients with advanced solid tumors, including HNC. OH2 was administered via an intratumoral injection under direct visualization or via ultrasound (US) guidance to deep-located nodes or organ metastases at viral doses of 10^6^, 10^7^, or 10^8^ cell culture infectious dose (CCID)_50_/mL. Of 54 patients, 40 received OH2 monotherapy, while 14 received combination therapy with OH2 and the anti-PD-1 antibody HX008. The median follow-up period was 5.41 months. Two cases of PR were observed in each group. There were no serious treatment-related AEs. The response rate increased with the addition of the anti-PD-1 antibody [40]. The seroprevalence of antibodies against HSV-1 was previously reported to be 66.6% in the 0–49 age group, while that of antibodies against HSV-2 was 13.2% in the 15–49 age group [21]. The intravenous administration of HSV-1 may result in viral inactivation by neutralizing antibodies. However, the use of oncolytic HSV-2, such as OH2, may allow for systemic administration of oncolytic HSV.

#### 3.1.2. Clinical Studies Using Oncolytic Adenoviruses for HNC

The first adenovirus used in the treatment of HNC was type 5, specifically ONYX-015, which lacks the E1B-55 kDa gene and, thus, exerts weaker inhibitory effects on p53 (Table 2). In 2005, Oncorine (H101), which has the same structure, was approved in China for HNC. In a study on recurrent HNC, the intratumoral administration of 2 × 10^11^ virus particles (VP) of ONYX-015 resulted in viral replication in tumor tissue, but not in the surrounding normal tissue. A tumor reduction ≧50% was observed in 21% of evaluable cases [41]. In another clinical trial, a group received 2 × 10^11^ VPs of ONYX-015 intratumorally for 5 consecutive days and the other group received twice-daily doses over 2 weeks; in the former group, 14% achieved PR-complete response (CR), 41% SD, and 45% PD, while in the latter group, 10% achieved CR, 62% SD, and 29% PD. Mild to moderate fever and pain were reported [46]. A phase 2 clinical trial was conducted combining ONYX-015 with cisplatin and 5-FU chemotherapy for recurrent HNC. Objective tumor control, including CR, was noted, and responding tumors showed no regrowth even after 6 months. In contrast, disease progression was detected in all cases treated with chemotherapy alone [47].

Group B adenovirus type 3 utilizes DSG2 as its primary high-affinity binding receptor and CD46 as its low-affinity receptor. Since antibody titers against group B adenoviruses are low in humans, the type 5/3 hybrid is less likely to be inactivated in the bloodstream than type 5, making it suitable for intravenous administration. TILT-123 is a hybrid adenovirus derived from types 5 and 3 (Ad5/3-E2F-d24) into which the TNF-α and IL-2 genes have been incorporated. It has the following structure: Ad5/3-E2F-d24-hTNFα-IRES-hIL2 [48]. In 2024, a phase 1 clinical trial on TILT-123 was conducted for advanced solid tumors, including HNC. Intravenous administration was performed on day 1, followed by intratumoral administration on days 8, 22, 36, 48, and 64. Doses for intravenous administration ranged from 3 × 10^9^ to 4 × 10^12^ VPs, while doses for intratumoral administration, ranged from 3 × 10^9^ to 5 × 10^11^ VPs in a dose-escalation scheme. At 78 days post-treatment, the disease control rate was 6/10 (60%) on PET, and 2/10 (20%) according to RECIST 1.1 and iRECIST. In cases with multiple tumors, shrinkage was observed even in tumors that did not receive intratumoral administration. There was no dose-limiting toxicity; however, fever, chills, and fatigue were reported. Therefore, TILT-123 is considered to be a safe oncolytic adenovirus that is effective against both local and distant lesions [42]. A patient with metastatic sinonasal mucosal melanoma was treated with TILT-123 and TILs. The virus was administered intravenously at a dose of 3 × 10^11^ VPs on the first day, followed by the administration of five doses of 1 × 10^11^ VPs into metastatic lymph nodes; the virus was not administered to the nasal cavity tumor. A single administration of TILs was performed intravenously. The case report describes a sustained, pathologically confirmed CR [49].

Since the enadenotucirev (ColoAd1) capsid is derived from Ad11p of the B group, CD46 and DSG2 expressed in many tumors were used as receptors. It has a large deletion in E3 and a small deletion in E4. Furthermore, it forms an Ad11p-Ad3 chimeric protein in the E2B region. The function of E1B-19K is impaired. These genetic modifications confer high stability to enadenotucirev in the bloodstream and the capacity for tumor-selective replication [50]. A phase 1 clinical trial was conducted to examine the combination of enadenotucirev and nivolumab for advanced or metastatic solid tumors, including HNC. Virus doses ranging from 1 × 10^12^ to 6 × 10^12^ VPs were administered intravenously. Among the 47 evaluable patients, the ORR was 2% and the SD rate was 45%. The one-year survival rate was 69%. Significant CD8^+^ T cell cytotoxic activity was observed in seven patients with CD8^+^ T cell infiltration within the tumor. Grade 3–4 treatment-related AEs, including anemia and infusion reactions, were noted in 31 of 51 patients (61%). Manageable AEs and promising therapeutic effects were reported in the trial [43].

NG-350A derived from enadenotucirev was developed by incorporating a full-length transgene for the anti-CD40 antibody downstream of the viral major late promoter. A phase 1a/Ib clinical trial was conducted using the intravenous and intratumoral administration of the virus in patients with metastatic or recurrent epithelial tumors. Viral detection in the blood persisted 7 weeks after the final dose, particularly in patients who received high-dose viral administration. The presence of the virus was also confirmed by tumor tissue biopsy [44].

VCN-01 is an Ad5-based oncolytic adenovirus engineered to replicate selectively in cancer cells with a dysfunctional RB1 pathway through the E1A Δ24 mutation and the insertion of an E2F-1 promoter upstream of E1A. It incorporates an RGDK motif in the fiber shaft to enhance integrin-mediated tumor targeting and expresses human recombinant hyaluronidase from the E3B region to degrade hyaluronan, thereby improving intratumoral viral spread and facilitating the extravasation of chemotherapeutic agents and immune cells into the tumor [51]. A phase 1 trial of intravenous VCN-01 and durvalumab (anti-PD-L1) was performed in patients with HNSCC refractory to immunotherapy. In the cohort receiving high dose (1.0 × 10^13^ VPs) followed by sequential durvalumab administration, the median OS was 17.3 months (95% CI, 8.1–NE). Among the sequential-administration cohorts, 7 of 14 patients (61.1%) survived for more than 12 months, which appears favorable compared with previously reported outcomes in anti-PD-(L)1–refractory HNSCC. Two dose-limiting toxicities (DLTs) occurred in the low-dose concomitant-administration cohort, and one DLT was observed in the low-dose sequential-administration cohort [7].

#### 3.1.3. Clinical Studies Using Oncolytic Vaccinia Virus for HNC

GL-ONC1 (GLV-1h68, Olvi-Vec) is a virus that lacks F14.5L (a secreted signal peptide) and A56R (hemagglutinin) in addition to J2R (TK). Furthermore, it contains genes encoding the Renilla luciferase-GFP fusion protein, β-galactosidase, and β-glucuronidase [52]. Preclinical studies demonstrated its antitumor effects in vivo against a wide range of tumors. Viral infection increased the number of cells in the S phase and sub-G1 phase and dose-dependently increased active caspase 3, indicating the induction of apoptosis in HNC cells [53,54]. In a phase 1 clinical trial, GL-ONC1 was added to cisplatin and radiotherapy for the treatment of advanced HNC patients (Table 2). The study included 19 patients who were p-16 negative, with 14 in stage IVA and 5 in stage IVB. The virus was administered intravenously at doses ranging from 3 × 10^8^ to 3 × 10^9^ PFU. The radiation dose was 70 Gy, and concurrent chemotherapy consisted of cisplatin at 100 mg/m^2^. The median follow-up period was 30 months. One- and two-year PFS rates were 74.4 and 64.1%, respectively, while one-and two-year OS rates were 84.6 and 69.2%, respectively. AEs related to GL-ONC1 included rigors, pyrexia, fatigue, hypotension, nausea, vomiting, and rash and pox-like lesions; there were no grade 4 toxicities related to the virus. GL-ONC1 was successfully administered intravenously in combination with chemoradiotherapy, yielding better treatment outcomes over historical values [45].

### 3.2. Clinical Study Using Oncolytic Viruses for Tumors Other than HNC

Although this review focuses primarily on HNC, the number of clinical studies using HSV, adenovirus, or vaccinia virus in HNC remains limited, and most are early-phase trials. Therefore, understanding how these viral platforms have been clinically applied in other solid tumors, such as melanoma, malignant glioma, colorectal cancer, liver cancer, and lung cancer, is essential for anticipating future developments in HNC. These tumor types represent the settings in which oncolytic virotherapy has progressed most rapidly, providing the most mature data on intratumoral and systemic delivery, dosing strategies, safety profiles, immune-modulating effects, and combination therapy. Accordingly, the clinical findings from these tumors are included not as part of the HNC disease category, but as indispensable reference models that inform the potential future application of virotherapy to HNC.

#### 3.2.1. Clinical Study Using Oncolytic HSV for Tumors Other than HNC

The clinical trial that served as the basis for the FDA approval of T-vec was conducted on melanoma [55]. In the 2019 final report of the OPTiM trial on unresectable stage III–IV melanoma, the median follow-up period was 49 months. ORRs were 31.5% in the T-vec group and 6.4% in the control group receiving GM-CSF, demonstrating the efficacy of T-vec [56]. PD-L1 expression was up-regulated in cells infected with HSV-1. Therefore, T-vec was combined with anti-PD-1 antibodies to block the PD-1/PD-L1 pathway. In advanced melanoma, a comparison between the group receiving T-vec in combination with pembrolizumab and the group receiving ICI alone showed ORRs of 48.6% in the combination group and 41.3% in the ICI group, with no significant difference [57]. In a phase 1 clinical trial on resectable acral melanoma, patients with stage III/IV disease received 12 weeks of neoadjuvant therapy with oncolytic HSV-1 orienx010, followed by 1 year of toripalimab as adjuvant therapy. All patients experienced grade 1–2 adverse reactions. At 2 years, recurrence-free survival was 81.5% and event-free survival was 73%, demonstrating a high response rate with neoadjuvant therapy [58]. When T-vec was used in combination with the anti-CTLA-4 antibody ipilimumab in advanced melanoma, ORRs for the combination and ipilimumab monotherapy groups were 35.7 and 16.0%, respectively. Five-year overall survival (OS) rates were 54.7 and 48.4%, respectively, demonstrating the sustained efficacy of combination therapy [59].

In addition to a double copy deletion of the ICP34.5 gene, G207 lacks the U39 gene of ICP6, which possesses RRfunction [60]. Clinical studies on G207 are being conducted for glioblastoma [61]. In a clinical trial targeting recurrent high-grade glioma in children, 10^7^–10^8^ PFU were administered over 6 h using intratumoral catheters. Median OS was 12.2 months, and 4 out of 11 patients were still alive 18 months later [62]. G47Δ is a construct that, in addition to ICP34.5 and ICP6 from G207, incorporates a deletion of ICP47. The phase 2 study involved the intratumoral administration of G47Δ to residual postoperative glioblastomas and recurrent tumors, with up to six repeat administrations. The one-year survival rate was 84.2%. Among 19 patients, two-year responses were 1 case of PR and 18 cases of SD [63]. Consequently, Teserpaturev (G47Δ) received conditional approval in Japan in 2021 for the treatment of recurrent and residual glioblastoma. CAN-3110 (formerly designated rQNestin34.5v.2) lacks two copies of the ICP34.5 gene and has a single copy of ICP34.5 inserted into the UL39 region under the control of the nestin promoter, enabling ICP34.5 expression in nestin-expressing tumors [64]. It was administered as a single intratumoral dose in a phase 1 clinical trial involving patients with recurrent high-grade glioma/glioblastoma. Median OS for the entire cohort was 11.6 months. There were no dose-limiting toxicities, or any cases of viral encephalitis or meningitis. This single intratumoral administration increased tumor-infiltrating lymphocytes (TILs) and changed the T-cell repertoire in both peripheral and tumor tissues, as well as gene expression within the tumor. These changes were more pronounced in patients who were positive for antiviral antibodies and led to higher survival rates [65].

T-vec and the anti-PD-L1 antibody atezolizumab were administered to patients with triple-negative breast cancer and liver metastases from colorectal cancer. T-vec was administered to liver lesions under US guidance. Overall response rate was 10% for breast cancer, while no efficacy was observed for liver metastases [66]. NV1020 is an oncolytic HSV derived from R7020, in which the L-S border region of HSV-1 has been replaced with that of HSV-2, and which incorporates exogenous TK gene regulated by an α4 promoter [67,68]. In a 1/2-phase clinical trial using NV1020 for liver metastases, the virus was used in combination with chemotherapy (oxaliplatin or irinotecan). Viral doses of 3 × 10^6^, 1 × 10^7^, 3 × 10^7^, and 1 × 10^8^ PFU were administered via hepatic artery infusion. Transient fever was observed. The one-year survival rate was 47.2%. The progression of liver metastases was halted with minimal AEs [69]. In a phase 1clinical trial on advanced sarcoma in adults, the intratumoral administration of T-vec at 1 × 10^8^ PFU/mL was combined with nivolumab and the alkylating anticancer agent trabectedin. The median follow-up period was 15.2 months. The one-year progression-free survival (PFS) was 36.7%. The confirmed best overall survival (RECISTv1.1) consisted of 3PR, 30SD, and 6PD. Grade 3 or higher AEs were observed in 50% of patients. This treatment has been evaluated as safe and effective [70]. In a study on intratumoral T-vec administration combined with pembrolizumab in patients with locally advanced or metastatic sarcoma, the ORR was 35% at the 24-week assessment, and antitumor activity was observed. AEs were mild [71].

#### 3.2.2. Clinical Studies Using Oncolytic Adenoviruses for Tumors Other than HNC

OBP-301 (Telomelysin) is an attenuated adenovirus type 5 into which the human telomerase reverse transcriptase promoter has been inserted to regulate the E1A and E1B genes. A phase 1 trial combining OBP-301 with radiotherapy was conducted on 13 patients with esophageal cancer. Patients received intratumoral injections of 10^10^, 10^11^, and 10^12^ VPs of OBP-301. Among the 13 patients, 8 achieved CR and 3 achieved PR, resulting in an ORR of 91.7%. Histologically, an increase in CD8^+^ T cells and up-regulated PD-L1 expression were observed [72].

TILT-123 was used to treat advanced solid tumors, primarily melanoma and ovarian cancer. A single intravenous dose of 3 × 10^9^ to 4 × 10^12^ VPs was administered. In 52 patients, post-treatment tumor biopsies revealed the presence of viral antigens and DNA, as well as changes in infiltrating immune cells. The median survival time differed depending on the presence or absence of viral infection in biopsy samples, at 280 and 190 days, respectively [73]. In a phase 1 trial on TILT-123 for metastatic melanoma, the ORR was 11.7% (2/17) and the disease control rate was 35% (6/17) [74].

In a phase 1 study on cisplatin-resistant ovarian cancer, enadenotucirev was used alone or in combination with paclitaxel; 1 × 10^12^ VPs were administered intravenously. With a median follow-up of 6.2 months, the 4-month progression-free survival (PFS) rate was 64%, ORR was 10%, and SD was 35% among 20 patients, and 65% experienced a reduction in target lesions at least once. Post-treatment biopsies showed increased CD8^+^ T cell infiltration. Treatment-related AEs of grade ≥ 3 were observed at least once in 63% (24/38) of patients [75].

DNX-2401 (tasadenoturev; Delta-24-RGD) lacks the 24 bases in the E1A gene that are involved in binding to Rb, and also incorporates an RGD motif into the fiber knob. Since an RGD-motif enables the virus to use αvβ3 or αvβ5 integrins highly expressed in cancer cells, the infection efficiency of tumor cells with low CAR expression has been improved. A phase 1/2 clinical trial combining the virus with pembrolizumab was performed for recurrent glioblastoma. Cannulas were inserted into the recurrent tumors of 49 patients, and 5 × 10^8^, 5 × 10^9^, and 5 × 10^10^ VPs were administered intratumorally; no dose-limiting toxicity was observed. The ORR was 10.4%, which did not statistically differ from the 5% in the prespecified control group. The 12-month survival rate was 52.7%, which was significantly higher than the 20% in the control group [76]. Ad-TD-nsIL12 is a type 5 adenovirus with three gene deletions (E1ACR2, E1B19K, and E3gp19K), while retaining E3B. Furthermore, it expresses non-secreting IL-12 (nsIL-12). In the treatment of recurrent high-grade glioma, 5 × 10^9^ to 5 × 10^10^ VPs were administered intratumorally. Grade 3 seizures were observed in two patients. Among the eight patients, one achieved CR and one achieved PR. The infiltration of CD4^+^ and CD8^+^ T cells into the tumor was observed. Safety and efficacy were suggested [77].

#### 3.2.3. Clinical Study Using Oncolytic Vaccinia Virus for Tumors Other than HNC

In a phase 1b clinical trial on GL-ONC1 for cisplatin-resistant ovarian cancer, GL-ONC1 was administered intraperitoneally at dose levels of 3 × 10^9^, 1 × 10^10^, and 2.5 × 10^10^ PFU. Among the 11 evaluable patients, the ORR was 9% (1/11), the SD rate was 64% (7/11), and 46% (5/11) achieved SD for ≥15 weeks. No grade 4 AEs were observed. The intratumoral infiltration of CD8^+^ T cells and the activation of tumor-specific T cells in peripheral blood were observed. The treatment was clinically safe and showed promise for efficacy [78].

JX-594 is an oncolytic virus, also known as Pexa-Vec, that lacks TK and expresses GM-CSF. In a phase 1b trial on 15 patients with colorectal cancer resistant to existing treatments, JX-594 was administered intravenously at doses of 1 × 10^6^, 1 × 10^7^, and 3 × 10^7^ PFU/kg. Ten patients (67%) achieved radiographic SD. There were no dose-limiting AEs [79]. The efficacy of combining JX-594 with durvalumab (anti-PD-L1) or tremelimumab (anti-CTLA-4) was also investigated in patients with metastatic colorectal cancer. The virus was administered intravenously at doses of 3 × 10^8^ or 1 × 10^9^ PFU. The median PFS in the JX-594/durvalumab/tremelimumab group and the JX-594/durvalumab group were 2.3 and 2.1 months, respectively. Although this treatment was safe, efficacy was not confirmed [80]. In a phase 2 clinical trial on soft tissue sarcomas, JX-594 was administered in combination with the PD-L1 inhibitor avelumab and the immunosuppressive agent cyclophosphamide. The dose of the virus injected into tumors was 1 × 10^9^ PFU. Among the 14 evaluable patients, one achieved PFS at 6 months. The most common AEs were fatigue and fever. An analysis of tissue biopsies and plasma samples revealed an increase in CD8^+^ T cell levels and the up-regulation of immune-related protein biomarkers, including CXCL10 [81].

TG6002 lacks the J2R (TK) gene and the I4L gene encoding the RR large subunit, and incorporates the fungal cytosine deaminase/uracil phosphoribosyltransferase fusion protein (FCU1) gene. The FCU1 gene product converts 5-fluorocytosine (5-FC) to 5-FU. A phase 1 clinical trial was conducted using TG6002 in combination with 5-FC to treat liver metastases from colorectal cancer. TG6002 was administered into the main hepatic artery at doses of 1 × 10^6^, 1 × 10^7^, 1 × 10^8^, and 1 × 10^9^ PFU. In 15 patients, an increase in serum 5-FU concentrations was observed. These findings suggested viral delivery via the hepatic artery, viral replication, and significant peripheral immune activation. However, clinical efficacy was not observed [82].

### 3.3. Platform-Specific Summary and Clinical Considerations of HSV, Adenovirus, and Vaccinia Oncolytic Viruses

When applying these three classes of oncolytic viruses to the treatment of HNC, the optimal viral agent should be chosen based on factors such as tumor location, size, degree of fibrosis and stromal density, and whether the disease is localized or accompanied by distant metastases. At the current stage of research, however, it remains difficult to clearly differentiate their suitability based solely on available evidence. HSV provides high flexibility for gene delivery and can accommodate large exogenous gene inserts, enabling localized expression of ICIsand cytokines directly within the tumor microenvironment [39]. Agents such as T-vec, which are already approved for melanoma and glioma, have demonstrated local efficacy and safety, and clinical trials using intratumoral injection are ongoing [62,63]. Owing to its strong infectivity, the required viral dose can be kept relatively low. Adenoviruses are also easy to genetically engineer, and numerous modified strains have been developed. They can replicate independently of the cell cycle [23]. Because disease control has been achieved through intratumoral viral delivery following intravenous administration, both routes are expected to be effective [7,44], although higher viral doses are generally required. Although clinical experience with vaccinia virus remains limited, it is considered promising for cases with distant metastases treated via intravenous injection, as well as for strategies aimed at systemic immune reprogramming [45]. At present, viral therapy is primarily indicated for recurrent or metastatic HNC that has failed to respond to standard treatments. In such settings, systemically deliverable viruses are appropriate. Nonetheless, because viral therapy is almost always combined with existing modalities, HSV may still play an important role in local tumor control through intratumoral administration. Given that individual HNC patients may respond differently to each viral platform, it is reasonable to maintain all three as viable therapeutic options.

To date, clinical trials have been limited to phase 1 and 2. Research remains constrained by small sample sizes, heterogeneous patient populations, early-phase trial designs, and the absence of randomized controlled studies. Moreover, because these trials typically enroll patients with progressive or recurrent disease, viral therapy is often administered alongside other treatments, making it difficult to isolate the antiviral effect and hindering advancement to later trial phases. As a result, drawing definitive conclusions about therapeutic efficacy remains challenging.

Clinical trials conducted in tumor types other than HNCprovide useful reference points for developing treatment strategies in HNC. Notable examples include the experience with intratumoral administration of HSV in melanoma and glioma [56,57,61,62]. In melanoma, when the virus is injected to adequately cover a localized lesion, clear therapeutic efficacy has been demonstrated, including high response rates with neoadjuvant therapy for resectable acral melanoma [58]. In brain tumors, repeated, continuous, and multidirectional viral delivery through a catheter, particularly targeting residual tumor at the resection margin, has produced therapeutic benefits. These findings offer valuable insight for designing local viral administration approaches in HNC. HSV has also been administered via hepatic arterial infusion in combination with chemotherapy for liver metastases. Because intra-arterial infusion increases the amount of virus reaching the tumor through the bloodstream, resulting in higher intratumoral concentrations [69], this strategy may also be applicable to HNC. In melanoma, no synergistic effect has been observed with the combination of T-vec and pembrolizumab (anti-PD-1), whereas improved efficacy has been reported with T-vec plus ipilimumab (anti-CTLA-4) [59]. These results underscore the challenge of selecting optimal ICIcombinations for HNC. For adenovirus-based therapies, studies have shown that a single intravenous injection can control advanced melanoma and ovarian cancer during initial treatment. Moreover, the presence or absence of viral antigens in tumor biopsies obtained after intravenous administration has a significant impact on prognosis [73,74], highlighting the importance of post-treatment biopsy evaluation. Intratumoral adenovirus administration is also being explored in brain tumors. In colorectal cancer, adding tremelimumab (anti-CTLA-4) to a regimen combining intravenous vaccinia virus therapy and durvalumab (anti-PD-L1) did not produce a clear improvement in progression-free survival [80]. These findings should be carefully considered when designing ICI combinations for HNC.

### 3.4. Preclinical Studies Using Emerging Oncolytic Viruses

#### 3.4.1. Preclinical Studies on Oncolytic HSV-1

Since one advantage of HSV is its ability to carry large foreign genes, modified viruses that are capable of expressing immunostimulatory cytokines have been developed. Clinical and pre-clinical studies with oncolytic HSV-1 that express cytokines, such as GM-CSF, IL-12, FLT3L, TNF-α, IL-2, IL-15, IL-18, CCL2, CCL5, and CXCL4, are underway [68]. An oncolytic HSV-1, OV-IL15C, expressing IL-15/IL-15Rα has been developed. When used in combination with chimeric antigen receptor-NK cells, it suppressed tumor growth and prolonged survival in mice with glioblastoma [83]. oHSV1-IL15B expresses IL-15/IL-15Ra and oHSV1-aPD-1 expresses an anti-PD-1 antibody. The combined effects of these two viruses on colorectal cancer were examined and a robust antitumor-specific T-cell response was observed [84]. Since the interaction between OX40 and OX40L leads to T-cell activation, OV-mOX40L expressing murine OX40L has been created. It increased tumor-infiltrating CD4^+^ and CD8^+^ T cells and reduced Tregs, leading to the prolonged survival of mice bearing pancreatic ductal adenocarcinoma [85].

Since molecules on the surfaces of tumors and immune cells may be the targets of oncolytic virus, HSV-1 targeting EGFR and PD-1 has been developed [86]. When HSV-1 is used intravenously, the clearance of the virus by the host’s phagocytic system in the bloodstream is a limitation. To overcome this issue, a recombinant virus FusOn-CD47-Luc that expresses CD47, a “do not eat me” signal molecule, on its surface was constructed. Systemic administration allowed the modified virus to survive for a longer time and be transported more efficiently to the tumor site. It exerted a measurable antitumor effect in a mouse tumor model, while the parental strain administered via the same route was ineffective [87]. Oncolytic HSV-2 strains expressing cytokines, such as IL-12, IL-15, GM-CSF, PD-1v, and IL-7×CCL19, have been created, and administered by intratumoral injection. In mouse models of breast and colorectal cancer, the strongest antitumor effects were observed when these virus strains were combined as a cocktail rather than being administered individually [88].

#### 3.4.2. Preclinical Studies on Oncolytic Adenoviruses

An adenovirus expressing an enhanced cross-hybrid IgGA Fc PD-L1 inhibitor was constructed. The cross-hybrid Fc-fusion peptide consists of an Fc region with the constant domains of IgA1 and IgG1, fused to a PD-1 ectodomain, thereby enabling binding to PD-L1. The oncolytic adenovirus has been shown to activate the Fc-effector mechanisms of IgA1 (neutrophil activation) and IgG1 (NK and complement activation), resulting in enhanced tumor killing over the anti-PD-L1 antibody atezolizumab [89]. Adenoviruses expressing anti-CTLA-4 antibodies have also been engineered [90].

In the treatment of solid tumors with chimeric antigen receptor T (CAR-T) cells, challenges include impaired tumor penetration and functional suppression within an immunosuppressive TME. Research is underway combining Ad5-ZD55-hCCL5-hIL12, which carries CCL5 and IL-12, with CAR-T cells targeting carbonic anhydrase 9. The oncolytic adenovirus increased CAR-T cell infiltration into tumor tissue and prolonged survival in mice with renal cell carcinoma [91]. While CAR-T cells targeting B7H3 alone did not suppress the growth of mouse glioblastoma, the antitumor effect was sustained by the intratumoral administration of oAd-CXCL11 armed with CXCL-11; CD8^+^ T cell infiltration increased, while MDSCs, Tregs, and M2 macrophages decreased [92]. A stable immune-activating ability was conferred to an adenovirus that expresses E1A downstream of the survivin promoter by expressing GM-CSF [93]. Tumor cells overexpress the membrane protein CD47 that binds SIRPα and send a “do not eat me” signal to evade immune elimination. The IgG2 anti-SIRPα antibody disrupts this binding. The adenovirus pDC316-oAd-SA was designed to express the SIRPα-mIgG1Fc gene, a fusion of the SIRPα and Fc genes, in order to remodel tumor-associated macrophages. In mouse models, this virus significantly enhanced macrophage phagocytic activity and exerted excellent tumor regression effects [94]. Hypoxic tumors express HIF-1 and exhibit resistance to treatment. To kill hypoxic/HIF-active tumor cells, the adenovirus HYPR-Ad-IL4 was constructed by cloning the E1A viral replication and IL-4 genes under the regulation of a bidirectional hypoxia/HIF-responsive promoter. This oncolytic adenovirus induced IL-4 expression, viral replication, and cytolysis in hypoxic cells, and suppressed tumor growth with a similar efficiency to that of wild-type *dl*309-Ad [95].

#### 3.4.3. Preclinical Studies on Oncolytic Vaccinia Virus

VV-iPDL1/GM, a vaccinia virus co-expressing a PD-L1 inhibitor and GM-CSF, has been reported. The oncolytic virus overcomes PD-L1-mediated immune suppression during both the priming and effector phases, inducing systemic T cell responses against neoantigenic epitopes derived from mutations. An intratumoral injection of the virus efficiently suppressed both virus-infected tumors and distant tumors [96]. Similarly, VV-α-TIGIT, a vaccinia virus that secretes a monoclonal antibody against TIGIT, has been engineered. An intraperitoneal injection of the vaccinia virus in an ascites tumor model achieved approximately 70% complete tumor regression. The increased recruitment of T cells and activation of T cells in TME were observed. When the same tumor was re-implanted into mice that had been cured by virotherapy, mice exhibited resistance, confirming the presence of tumor-specific immune memory [97].

To improve the safety of vaccinia virus for non-tumor cells, modifications are made to delete non-essential genes. WR-Δ4 lacks the following four genes, J2R (TK), A48R, B18R (soluble type I interferon receptor), and C11R (VGF). These deletions affect the metabolism, proliferation, and signaling pathways of infected cells. When the modified vaccinia virus was administered intraperitoneally to mice with melanoma, the virus’s virulence and spread were suppressed, and tumor growth was effectively inhibited [98]. Since vaccinia virus accommodates large payloads, viruses have been engineered to incorporate various interleukin genes, including IL-2, IL-10, IL-12, IL-15, IL-21, IL-23, IL-24, and IL-36γ. This engineering of virus is expected to facilitate the recruitment of immune cells, control immune suppression, and enhance systemic antitumor immunity [99]. To recruit effector T cells, a vaccinia virus that expresses CXCL11, a ligand for CXCR3, has been constructed. Following an intravenous injection of the virus (VV.CXCL11) and mesothelin-specific CAR-T cells, an increase in tumor-specific T cells and enhanced antitumor immunity were observed in mesothelin-expressing tumors [100]. CD55, also known as the decay-accelerating factor, is a protein that locates on the cell surface that protects the cell from complement-mediated attack. SJ-607 is a vaccinia virus expressing human CD55 protein and GM-CSF. In a human cancer xenograft model, it expressed CD55 and exhibited higher antitumor activity than JX-594 [101].

## 4. Challenges and Strategies in Oncolytic Virotherapy for HNC

The intratumoral administration of an oncolytic virus has been the primary approach, as exemplified by T-vec for solid tumors. However, in cases of large tumors or recurrence, it is difficult to cover the entire tumor with local administration. Uniform viral diffusion throughout the tumor may be limited by physical barriers, such as tumor compactness, tumor heterogeneity, and high pressure within the TME [102]. On the other hand, systemic administration via an intravenous injection also presents challenges, including the need for higher doses, inactivation of the virus by antibodies, binding to blood components, adhesion to blood vessels, and accumulation in the liver and spleen. This section explains the efforts to overcome these challenges.

### 4.1. Modification of the Surface of Oncolytic Viruses

To prevent the inactivation of oncolytic viruses by neutralizing antibodies in blood vessels, specific viral strains are selected, such as using HSV-2 instead of HSV-1, adenovirus type 3 instead of type 5, and the vaccine strain of a vaccinia virus. However, the repeated systemic administration of viruses leads to the production of new antibodies, which reduce the virus’s infectivity. Nanomedicine research is being conducted on modifications to oncolytic viruses [103]. Due to their small size and lack of an envelope, adenoviruses are frequently used for their surface modifications, using liposomes, polymers, and albumin [104]. Many modified viruses, including a polyethylene glycol (PEG)-conjugated adenovirus, PPSA polymer-coated adenovirus, DA3 polymer-coated adenovirus, PEG-*b*-PHF polymer-coated adenovirus, ABP polymer-conjugated adenovirus, and chitosan-coated adenovirus have already been developed. These viruses showed an extended circulation time and were efficient against disseminated metastatic lesions [105].

Research on PEG conjugation has been conducted with HSV-1. Since folate receptors are highly expressed in HNC, G207 was covalently modified on its surface with a folate-PEG conjugate to reduce viral immunogenicity and target tumor cells expressing the folate receptor. When the modified virus, FA-PEG-HSV, was intravenously administered to nude mice with KB cell xenografts, high antitumor activity was observed [106]. An oncolytic HSV-1 modified with galactose-PEG has recently been created. Electron microscopy of the parent virus revealed an average diameter of 227.6 nm, while glycosylated-PEG-oHSV-1 measured 247.2 nm. The modified HSV-1 specifically binds the asialoglycoprotein receptor, which is highly expressed in hepatocellular carcinoma (HCC). The intravenous administration of the virus promoted the infiltration of activated T cells and NK cells, stimulated the secretion of antitumor cytokines, and inhibited tumor progression. The production of antiviral neutralizing antibodies was suppressed [107]. Surface modifications make it possible to deliver HSV-1 systemically.

### 4.2. Transport of Oncolytic Viruses Using Cellular Carriers

The ideal characteristics required of carrier cells used for systemic virus delivery include the ability to migrate toward the TME, susceptibility to viral infection, and the ability to survive long enough to reach the tumor site and release the virus. The cells being studied primarily include mesenchymal stem cells (MSCs), T cells, and hematopoietic cells [108,109,110]. MSCs infected with oncolytic HSV-1 efficiently reached metastatic tumors, and the virus was detected within tumors. As a result, survival rates of the treated mice with brain tumors were prolonged [111]. HSV-1 R3616 was adsorbed by tumor-antigen-specific lymphocytes derived from mice that acquired antitumor immunity. When mice carrying tumors in the peritoneal cavity were treated with virus-adsorbed lymphocytes intraperitoneally, they survived significantly longer than mice that simultaneously received non-specific lymphocytes and the virus [112]. CAR-T cells are expected to be used as carriers of oncolytic viruses, yielding a dual effect, the cytotoxicity of T cells and the oncolytic activity of the virus. B7-H3 CAR-T cells infected with HSV^dko^-GFP were administered intravenously to mice with glioblastoma. This led to viral infection and T-cell infiltration in the tumor, and significantly prolonged the survival of mice [113].

The findings of a clinical study with adenovirus-infected autologous MSCs were reported. The study included 16 pediatric and adult patients with advanced cancer. Patients received cell doses of 2 × 10^6^ cells/kg or 0.5–1 × 10^6^ cells/kg over a 6-week period. Each cell was infected with 2 × 10^4^ VPs. SD was observed in two patients with glioblastoma, one of whom maintained this status for 6 weeks. No grade 2–5 AEs were observed [114]. However, carrier cell preparation is time-consuming, and challenges regarding whether the virus will be released at the tumor site and successfully infect target cells still remain [103].

### 4.3. Research on the Local Administration of Oncolytic Viruses

When adult patients have antibodies against a virus, such as HSV-1, the oncolytic virus is typically injected into tumors. Intratumoral administration is well tolerated because the drug infiltrates the injection site, achieves high local tissue concentrations, and is gradually absorbed. The injected virus may quickly reach local lymph nodes, which is also advantageous for initiating and maintaining an immune response [115]. The intratumoral administration of an oncolytic virus is currently the most reliable method for primary lesions of melanoma, brain tumors, and HNC. In a previous study on HNC, oncolytic HSV-1 was administered to metastatic cervical lymph nodes [34]. Administration into deep-seated tumors may be performed under image guidance [116]. Regarding brain tumors, a catheter is placed to allow for prolonged viral administration [62,63].

High-pressure needle-free administration systems have been reported to distribute the virus more uniformly subcutaneously and intramuscularly than injections [117]. This approach may be useful in cases of superficial tumors, such as SCC or melanoma arising in the mucosa covering the bone like the hard palate. Hydrogels exhibit excellent biocompatibility and sustained drug release kinetics. They also do not decrease viral infectivity. An adenovirus carrying the CRB3 and GM-CSF genes was encapsulated into a hydrogel, resulting in Adv-CRB3@gel. When it was administered into bladder cancer in mice, cancer proliferation was suppressed, and the infiltration of immune cells into tumor tissue increased. Its combination with a PD-L1 antibody further enhanced this effect [118]. A hydrogel also acted as a delivery reservoir of oncolytic HSV-1. When locally administered to HCC, it maximized viral replication and intratumoral distribution, resulting in prolonged disease-free survival and reduced tumor recurrence in animals [119].

### 4.4. Application of Microbubbles (MBs) and US for the Delivery of Oncolytic Viruses

**Figure 1:**
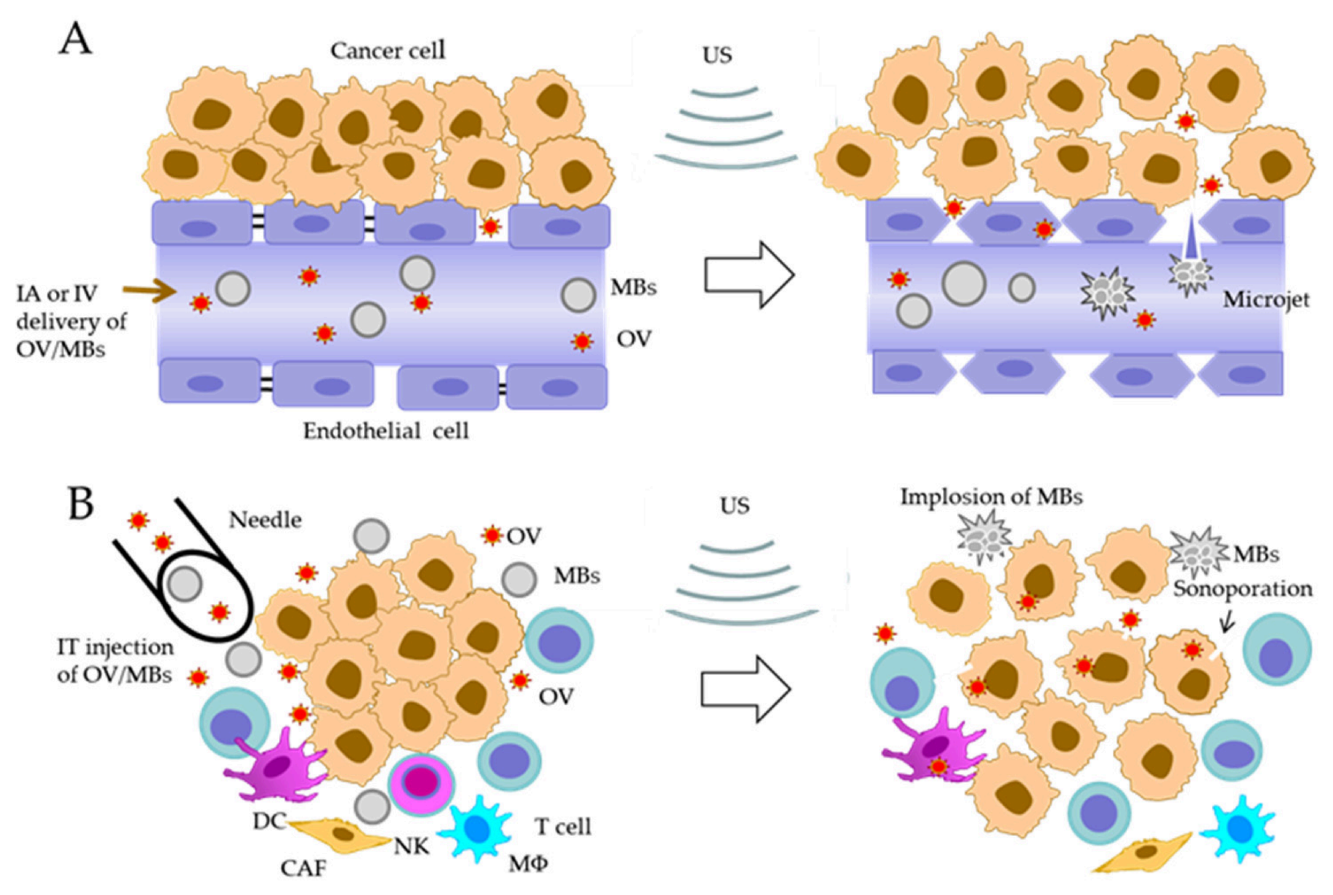
Illustration of the OV delivery mechanism via MBs/US. (**A**) MBs and OV are delivered to tumors via blood vessels. Under US irradiation, MBs oscillate in a stable manner, generating microfluidic jets, or implode violently, producing shock waves and high-velocity microjets. This force promotes the extravascular infiltration and migration of OV within the TME. (**B**) Injecting MBs and OV into the tumor and subsequent applying US induces a physiological shearing stress and sonoporation, promoting viral diffusion within the TME and the entry of OV into tumor cells. IA, intra-arterial; IV, intravenous; IT, intratumoral; OV, oncolytic virus; MBs, microbubbles; US, ultrasound; DC, dendritic cell; CAF, cancer-associated fibroblast.

To induce viral oncolysis, it is necessary to promote the extravasation of systemically administered viruses and the intercellular diffusion of viruses. The combination of MBs and US is a useful method for the delivery of antitumor agents [120,121,122]. In this method, when MBs are administered intravenously and reach the target tumors, they are exposed to US. At low acoustic pressures, MBs oscillate in a stable manner (stable cavitation), generating microfluidic jets. At high acoustic pressures, MBs oscillate violently and implode (inertial cavitation), producing shock waves and high-velocity microjets. These forces apply shearing stress to cells, reorganize tight junctions, activate endocytosis pathways, and induce pore formation in the cell membrane, a process known as sonoporation [123,124]. The diameter of membrane pores is 30–100 nm, and pore formation has been reported to persist for several seconds to minutes. Since this effect is limited to the area where US is applied, systemic toxicity caused by drug or viruses is minimized [123] (Figure 1A). In clinical settings, after the intravenous injection of temperature-sensitive liposomes encapsulating doxorubicin, focused US was applied to the target liver, resulting in a 3.7-fold increase in the drug concentration within the tumor [125].

The adenovirus Ad5/3-C-RGD-D24 was mixed with lyophilized MBs during thawing and MB and oncolytic virus resuspension was created, which was tested in a humanized mouse model of triple-negative breast cancer. Three groups were established: an intratumoral injection, intravenous injection, and intravenous injection followed by US irradiation. When the percentage of virus antigen-positive cells was measured 4 weeks later, it was 50–80% for the intratumoral injection group, 1–5% for the intravenous injection group, and 50–75% for the intravenous and US groups, demonstrating that the virus was successfully transported using MBs and US [126]. In a prostate cancer model, Ad.PEG-E1A-*mda*-7 and MBs were administered via the tail vein, followed by US irradiation of the tumor. This treatment resulted in a marked reduction in the tumor burden in mice with xenografts [127].

On the other hand, in an in vitro system, HNC cells were exposed to US in the presence of oncolytic virus and MBs, which increased viral uptake into cells without affecting cell viability [128]. When MBs and oncolytic HSV-1 were directly injected into HNC tumors in nude mice followed by US irradiation, the number of virus antigen-positive tumor cells increased and the antitumor effect became more pronounced than with the virus injection alone [129]. This was presumed to be because the injected viruses entered tumor cells via sonoporation within a short time in addition to receptor-mediated pathway (Figure 1B). MBs and US irradiation may expand intercellular spaces and the extracellular matrix, which restrict viral diffusion, thereby promoting viral spread. This method may also affect the biological activity of existing non-tumor cells, such as macrophages, Tregs, MDSCs, cancer-associated fibroblasts, and endothelial cells in the TME [108,130].

### 4.5. Studies on Promoting the Replication of Oncolytic Viruses Within Tumor Cells

Even when oncolytic viruses are delivered to tumor cells, they do not necessarily replicate and produce large numbers of progeny viruses. Consequently, research on drugs to enhance viral replication are required. One well-studied compound is the histone deacetylase (HDAC) inhibitor [131]. HDACs remove acetyl groups from histone residues, allowing negatively charged DNA to bind nucleosomes and act to suppress transcription. HDAC inhibitors regulate the chromatin structure and gene transcription and induce a diverse range of biological responses in tumors. In combination with oncolytic viruses, HDAC inhibitors have been reported to suppress the transcription of IFN-responsive genes, induce NF-κB signaling and autophagy, enhance viral receptor expression, halt viral clearance by the innate immune system, and enhance antitumor adoptive immune responses [131,132,133]. The HDAC inhibitor valproic acid (VPA) enhanced the replication of HSV-1 encoding GM-CSF, the production of GM-CSF, and oncolysis, and boosted the development of antitumor immunity. Mechanistically, VPA increased the expression of activating ligands for NK cell recognition and induced tumor antigens, thereby enhancing the cytotoxic activity of NK cells and the priming of CTLs [133]. Among several HDAC inhibitors, trichostatin A was found to potently promote the replication of oncolytic vaccinia virus in vaccinia virus-resistant cancer cell lines. The combination of trichostatin A and vaccinia virus improved survival rates in mice with xenografts of HCT colon tumors [134]. Trichostatin A also increased viral replication in oncolytic HSV-1, while reducing the proliferation of HNC cells [135]. Furthermore, trichostatin A enhanced the cell fusion of tumor cells infected with the fusogenic vaccinia virus FUVAC. In vivo, it increased the antitumor activity of FUVAC and prolonged the survival of mice carrying TC26 colon cancers [136]. VPA enhanced CAR expression in human lung cancer cells, improving the efficiency of the oncolytic adenovirus OBP-301 infection in all four lung cancer cell lines examined [137]. Dimethyl fumarate (DMF) is an HDAC modulator used to treat multiple sclerosis and psoriasis. A pre-treatment with DMF reduced cell viability in several cancer cell lines, including melanoma, while it significantly increased the oncolytic activity of HSV-1. This enhancement of oncolytic activity was attributed to the suppression of type I interferon production and responses [138].

Indoleamine 2,3-dioxygenase 1 (IDO1) is an immunosuppressive protein that is highly expressed in many tumors; it catalyzes the conversion of tryptophan to kynurenine [119,139]. The infection of HCC cells with GFP-tagged HSV-1 increased IDO1 expression, decreased tryptophan levels, and inhibited protein synthesis. However, the IDO1 inhibitor navoximod increased HSV-1 replication and enhanced virus-mediated tumor lysis. The administration of navoximod to HCC-bearing mice prolonged survival times and protected mice against tumor recurrence [119]. Cisplatin enhances the expression of GADD34, which is induced in response to cellular stress. Due to its structural similarity to ICP34.5, GADD34 binds to PP1 and dephosphorylates eIF2α, thereby lifting the inhibition of protein translation. Consequently, cisplatin promotes the replication of oncolytic HSV-1 [140]. Rapamycin, an mTOR inhibitor, is ineffective against tumor cells in which viruses fully replicate; however, in semi-permissive cells, such as EC9706 (esophagus), MCF-7 (breast), and Hela (cervix) cells, it strongly promotes viral production and spread. Furthermore, it inhibited or reduced the growth of EC9706 xenograft tumors [141].

## 5. Treatment of HNC Using Non-Replicating Viruses

The most advanced approach in the clinical management of HNC is currently immunotherapy using ICIs [142]. Studies are underway to evaluate ICIs either as monotherapy or in combination with chemotherapy. Meanwhile, since EBV is involved in the pathogenesis of nasopharyngeal cancer and HPV in that of oropharyngeal cancer, therapeutic vaccines targeting viral antigens are being tested as immunotherapy. The targets are the EBV antigens EBNA/LMP2 and HPV16 antigens E6/E7 [143,144,145]. When viruses are not involved in the etiology, the targets for cancer vaccines are TAAs and TSAs. Examples of TSAs include mutant TP53, while those of TAAs include MAGE-A3, YE-NEO-001, carcinoembryonic antigen, and Mucin 1 (MUC1) [146,147]. Virus vectors enter DCs and macrophages, where they express tumor antigens that are expressed via MHC class I and MHC class II pathways, thereby inducing robust innate and adaptive immune responses [147]. Unlike oncolytic virotherapy, virus for cancer vaccines does not replicate and stably expresses antigens through its transcription and translation functions.

### 5.1. Gene Expression Using Non-Replicating Adenoviruses and Cancer Vaccines

One form of virotherapy involves inserting cytokine genes into non-replicating adenoviruses and inducing their expression in vivo. AdGV.EGR.TNF.11D is a non-replicating adenovirus vector in which the TNF-α gene is linked to a radiation-responsive promoter. It produces TNF-α within the tumor in response to radiation and avoids the systemic toxicity of TNF-α. A phase 1 clinical trial was undertaken on patients with recurrent HNC receiving radiotherapy. These patients received the intratumoral administration of AdGV.EGR.TNF.11D (4 × 10^9^–4 × 10^11^ particle units) in combination with chemotherapy using 5-FU and hydroxyurea. Among 14 patients, the response rate was 83.3%, median survival was 9.6 months, and one patient survived for more than 3 years after treatment [148]. Additionally, in a phase 2 clinical trial on 136 patients with advanced HNC, the non-replicating adenovirus E10A, which expresses the angiogenesis inhibitor endostatin, was administered intratumorally in combination with cisplatin and paclitaxel. The ORR with the addition of the virus was 29.9%, which was not significantly different from the 39.7% observed with chemotherapy alone; however, patients treated with endostatin had longer PFS (7.03 versus 3.60 months). The overall disease control rate improved from 80.6% in the control group to 92.6%. There were no additional AEs other than fever [149].

In a phase 2 trial on EBV-associated nasopharyngeal carcinoma, a vaccine consisting of autologous DCs transduced with LMP1 and LMP2 via an adenovirus was injected intradermally. No significant AEs developed in 16 patients with metastatic disease. Clinical responses were observed in three patients. Although efficacy was limited, safety was confirmed [150]. A non-replicating adenovirus that expresses TAAs was used in clinical trials. Three adenoviruses ETBX-011, ETBX-061, and ETBX-051, which express CEA, MUC1, and brachyury, respectively, have been combined as the Tri-Ad5 vaccine. In newly diagnosed HPV-negative advanced HNC, the Tri-Ad5 vaccine (injected subcutaneously) was used in combination with bintrafusp alfa (anti-PD-L1 and anti-TGF-β) as a neoadjuvant therapy. Clinical-to-pathological downstaging was observed in two of the six patients with HNC (33.3%). Although CRs were not achieved, the two-year recurrence-free survival rate was 83.3%, which was more favorable than historical values [151].

### 5.2. Cancer Vaccines Using Non-Replicating Vaccinia Virus

A modified vaccinia virus Ankara (MVA) was developed as a smallpox vaccine; its safety has been demonstrated through long-term administration. The virulence of vaccinia virus stems from its ability to replicate. However, MVA does not cause skin reactions because it cannot replicate in human cells. Research on MAV-mediated cancer vaccines to deliver tumor antigens is underway [152].

Although TP53 mutations have been reported in approximately 50% of human cancers, the p53 pathway is considered to be dysfunctional in nearly all tumors [153]. Most of the p53 epitopes presented on the surface of tumor cells with TP53 mutations are the wild type and are recognized by T cells. If an immune response to p53 is present, it acts as a cancer antigen. Consequently, clinical studies are underway using MVA expressing wild-type p53 as a cancer vaccine. A phase 1 clinical trial using p53MVA with pembrolizumab was undertaken for advanced solid tumors, including HNC. Even after 49 weeks, 3 of 11 patients achieved SD, and an increase in and the maintenance of p53-reactive CD8^+^ T cells were observed in 2 patients, confirming the safety and clinical utility of the treatment [154].

TG4010 is an MVA that incorporates genes encoding MUC1 and IL-2. A phase 2b/3 clinical trial on subcutaneous TG4010 was conducted on patients with advanced non-small cell lung cancer expressing MUC1. Of 222 patients, 50% received TG4010 (10^8^ PFU) in combination with chemotherapy, while remainder received chemotherapy alone. Median PFS were 5.9 and 5.1 months, respectively, indicating a slight improvement in prognosis. No serious AEs related to TG4010 were noted [155]. A phase 1 clinical trial on TG4050 for HNC was recently undertaken. TG4050 is an MVA that incorporates the gene sequences of more than 30 individualized neoantigens predicted in silico for each patient. This vaccine was administered subcutaneously to patients with HPV-negative stage III or IV HNC who underwent surgery and received adjuvant chemoradiotherapy. The total number of doses was 20. All 16 patients treated with TG4050 as adjuvant immunotherapy remained free of recurrence after a median follow-up of 24.1 months. In contrast, among the patients in the observation control group, 3 out of 16 experienced recurrence [156]. These findings indicate that TG4050 induces tumor-specific adaptive immunity and prevents recurrence in locally advanced HNC.

## 6. Prospects of Virotherapy for HNC

**Figure 2:**
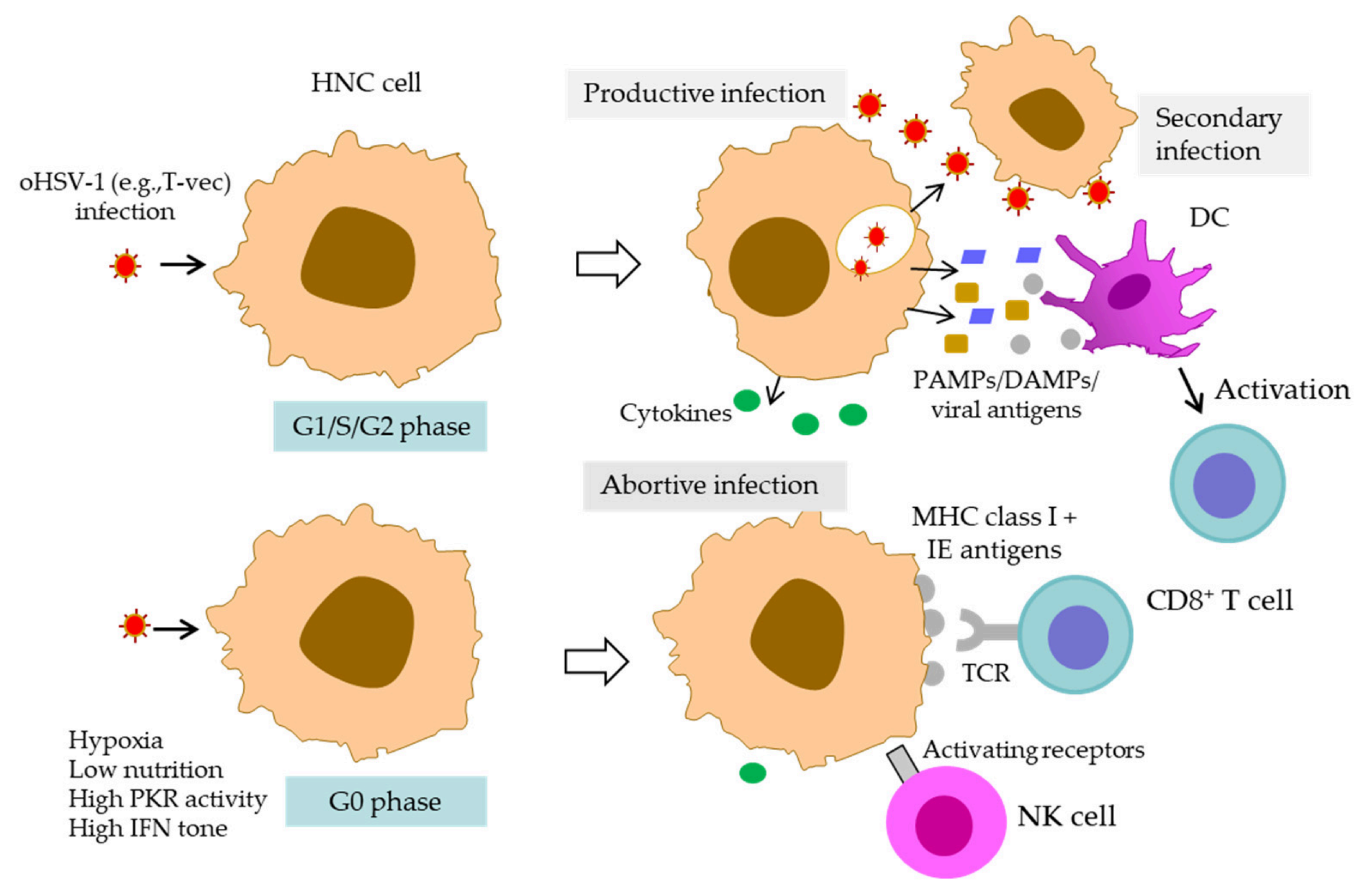
Illustration of oncolytic HSV-1 infection and the antiviral cellular immune response in heterogeneous tumor cells of HNC. HNC cells in the G1/S/G2 phases permit the replication of the virus, leading to immunogenic cell death. This results in the release of cytokines, progeny viruses, PAMPs, DAMPs, and viral antigens. DCs present viral antigens to CD8^+^ T cells. In quiescent G0-phase cells, viral replication is not permitted, resulting in abortive infection. These cells still express immediate-early genes of oncolytic HSV-1 and serve as targets for NK cells and activated CD8^+^T cells. HNC, head and neck cancer; oHSV-1, oncolytic HSV-1; PAMPs, pathogen-associated molecular patterns; DAMPs, damage-associated molecular patterns; PKR, double-stranded RNA-dependent protein kinase; IE, immediate-early; TCR, T cell receptor.

The replication of oncolytic virus induces the immunogenic cell death of tumor cells and the release of immunostimulatory molecules into the TME. Oncolytic virus carrying genes such as GM-CSF, IL-12, IL-15, and PD-1 antibody also express these foreign genes, thereby enhancing anti-tumor immunity within a tumor. However, it remains unclear whether oncolytic virotherapy induces tumor cell lysis across a wide area of a tumor. Quiescent tumor cells at the G0-phase may appear under conditions of hypoxia, malnutrition, and high IFN activity. In these cells, viruses such as oncolytic HSV-1 cannot replicate, resulting in abortive infection, while cells at the G1/S/G2 phase allow viral replication, leading to productive infection. These different types of infection, being dependent on the cell-cycle, may coexist in HNC (Figure 2). This is one reason why the sufficient tumor shrinkage of HNC was not achieved when T-vec was administered intratumorally [35]. Nevertheless, cellular immunity that typically targets the glycoproteins of the HSV-1 envelope also recognizes immediate-early proteins, such as ICP4 and ICP27, expressed on quiescent tumor cells. These tumor cells may be the target of antiviral immunity, even in abortive infection, although the magnitude of the response is decreased [157,158]. Antitumor cytotoxic T cells targeting tumor antigens destroy tumor cells irrespective of viral infection. Therefore, it is crucial to deliver as many viral particles as possible to tumor cells and ensure their infection.

In cases of maxillary cancer near the orbit, post-operative scarring and atrophy over time may cause significant facial deformity, resulting in major functional and esthetic impairments. Difficulties are associated with administering oncolytic viruses locally to tumors such as those in the maxilla. Intra-arterial infusion may achieve higher tumor concentrations even with lower viral doses. A phase 2 clinical trial, involving the intra-arterial infusion of the adenovirus ONYX-015 was undertaken for liver metastases from colorectal cancer. The virus was administered at a dose of 2 × 10^12^ VPs into the hepatic artery [159]. Similarly, in a phase I clinical trial on liver metastases, HSV-1 NV1020 was administered into the hepatic artery [160]. In cases of maxillary cancer nourished by the maxillary artery, intra-arterial infusion presents a well-known approach for the effective delivery of chemotherapeutic drugs [161]. This method involves advancing a catheter to the maxillary artery either from the femoral artery in the groin or from the superficial temporal artery in the temporal region [162,163]. In a clinical trial, this method was applied to oral cancer patients; a catheter was placed retrograde via the superficial temporal artery and served as the administration route of rAd-p53, a non-replicating adenovirus, and chemotherapeutic drugs. Survival rates were higher in stage III patients receiving the combined approach than in the group receiving chemotherapy alone; however, no significant difference was observed in stage IV patients [164]. The lingual artery and facial artery are also potential routes for intra-arterial infusion.

The route of administration for oncolytic viruses is selected based on tumor accessibility and therapeutic intent. Intratumoral injection delivers high concentrations of virus directly to superficial or easily accessible lesions and is suitable for small to moderate-sized tumors. Intravenous administration enables systemic delivery for widespread disease or distant metastases; however, given the inactivation of the virus in circulation, high doses are required, and acute toxicity must also be considered. Intra-arterial infusion allows high local viral concentrations while minimizing neutralization by circulating antibodies, but it is limited by the need for vascular access and anatomical suitability. Vaccination (subcutaneous or intradermal) aims to induce systemic antitumor immunity.

**Figure 3:**
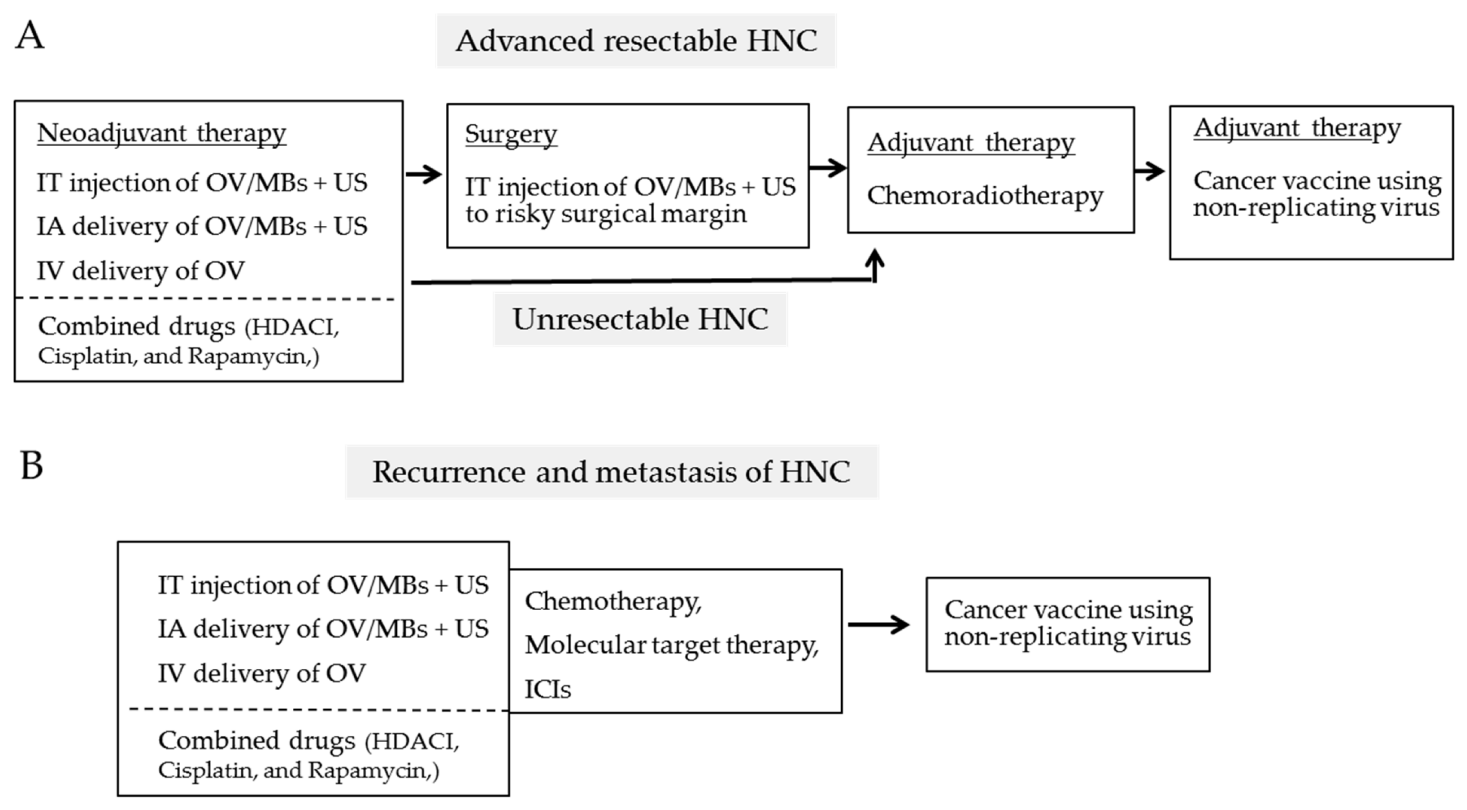
Proposal of virotherapy for advanced HNC. (**A**) In cases of resectable HNC, an OV is administered locally and systemically as a neoadjuvant therapy to reduce the tumor burden. MBs/US with or without drugs that promote viral replication may be used. Following surgery, adjuvant chemoradiotherapy is administered if risk factors are identified. Cancer vaccines using non-replicating viruses are useful for preventing tumor recurrence. (**B**) OV/MBs/US is also used in combination with conventional therapies to treat locally recurrent tumors. Treatment for metastatic lesions is possible via the intravenous administration of oncolytic viruses. IT, intratumoral; IA, intra-arterial; IV, intravenous; OV, oncolytic virus; MBs, microbubbles; US, ultrasound; HDACI, histone deacetylase inhibitor; ICIs, immune checkpoint inhibitors.

A characteristic of virotherapy is that, while the frequency of AEs is high, these events do not result in the type of long-term AEs that affect quality of life. Therefore, it may be used as a neoadjuvant therapy in resectable cases or as a pre-treatment for radiotherapy. As a proposed treatment plan, if there is a tumor detectable by a visual examination or imaging, the first step is to reduce the tumor volume using oncolytic viruses. This approach is supported by the findings of preoperative studies using T-vec in regional lymph nodes for HNC, as well as neoadjuvant therapy with oncolytic virus for colorectal cancer, pancreatic cancer, and melanoma [34,58,165,166]. An oncolytic virus is administered intratumorally for resectable HNC; it needs to be injected slowly over time, changing the injection site as necessary. Additionally, oncolytic viruses, which are resistant to inactivation in the bloodstream, may be administered systemically via an intravenous injection. If applicable, intra-arterial administration is useful for delivering high doses of an oncolytic virus to the wide area of a tumor. MBs/US (with or without viral replicators) may be applied for the local and intra-arterial delivery of an oncolytic virus. After surgery, adjuvant chemoradiotherapy is required if risk factors are identified (Figure 3A). In unresectable cases, treatment is performed in a similar manner without surgery. In cases of recurrence, oncolytic viruses are administered in combination with conventional therapies such as chemotherapy, molecular targeting therapy, and ICIs. In a study on adenovirus TILT-123 for cases of multiple solid tumors, the intravenous and intratumoral administration of the virus resulted in the shrinkage of many tumors, suggesting the effectiveness of an intravenously administered adenovirus for both local and distant lesions [42]. Therefore, it is desirable to intravenously administer oncolytic viruses for distant metastasis (Figure 3B). The MVA-based cancer vaccine TG4050 has been shown to suppress recurrence in locally advanced HNC [156]. Virus-based cancer vaccines may be applied to prevent recurrence.

Although robust clinical evidence for oncolytic virus therapy in recurrent or metastatic HNC remains limited, the treatment strategy proposed here is also based on information that includes clinical findings from solid tumors, such as melanoma, malignant glioma, colorectal cancer, and pancreatic cancer, where the efficacy of oncolytic viruses has been demonstrated in both intratumoral and systemic administration. However, much of this evidence is derived primarily from phase 1/2 clinical trials rather than phase 3 trials, and findings regarding HNC are limited. Therefore, the treatment plan shown in Figure 3 should be interpreted as a future prospect rather than an established clinical recommendation. In summary, current clinical studies demonstrate that oncolytic viruses are feasible and immunologically active in HNC, although their therapeutic impact remains modest. Clinical experience from various solid tumors provides important insights into optimal delivery routes, dosing strategies, and combination approaches that may be applicable to HNC. Key challenges specific to HNC include improving viral penetration into bulky or fibrotic tumors, overcoming rapid viral inactivation in the bloodstream, and identifying biomarkers that predict response. Future research should focus on refining delivery methods (including biomaterials, nanoparticles, and exosomes), engineering next-generation viral vectors (AI-aided design, genome editing), and designing rational combination regimens to maximize antitumor immunity. These considerations form the basis of the conceptual treatment framework proposed in this review.

## 7. Conclusions

In the field of virotherapy, research using oncolytic viruses is advancing. We are now at the stage where the findings of registered clinical trials are being published. Representative examples of DNA viruses include HSV, adenoviruses, and vaccinia virus. Among these, adenoviruses have been the subject of extensive research due to the ease of their genetic manipulation, their ability to replicate independently of the cell cycle, the possibility of systemic administration, and their utility as a vaccine. However, a number of challenges remain, such as their accumulation in the liver, binding to blood components, receptor expression, and the need for high viral loads. In HNC, recurrent and metastatic lesions have long been the targets of virotherapy. A major characteristic of virotherapy is that, while acute AEs occur, they do not result in permanent disabilities, such as those that occur with surgery or radiotherapy. Furthermore, the antitumor activity of an oncolytic virus relies on virus-replication-induced immunogenic cell death, which is largely independent of the canonical mechanisms underlying resistance to platinum-based chemotherapy and radiotherapy. Therefore, oncolytic viruses may be utilized as neoadjuvant or adjuvant therapy for surgery and radiotherapy. Cancer vaccines based on non-replicating viruses may be useful for preventing recurrence. It may be argued that virotherapy needs to be incorporated into conventional treatment and utilized throughout the entire course of therapy.

## Figures and Tables

**Table 1:** Basic properties of herpes simplex virus, adenoviruses, and vaccinia virus in virotherapy.

Virus	Herpes Simplex Virus	Adenoviruses	Vaccinia Virus
Genome	dsDNA 150 kb	dsDNA 36 kb	dsDNA 190 kb
Size	100–200 nm	90–100 nm	270 × 350 nm
Genome capacity	30–40 kb	7–8 kb	25–40 kb
Virion	Enveloped	Naked	Enveloped
Main virus components binding to receptors	gB, gC, gD, gH, gL	Fiber knob	D8L, H3L, A27L
Main cellular receptors	HVEM, Nectin-1,Nectin-2, Heparan sulfate	CAR, CD46, DSG2	Chondroitin sulfate, Heparan sulfate
Replication site	Nucleus	Nucleus	Cytoplasm

HVEM, herpes virus entry mediator; CAR, coxsackievirus and adenovirusreceptor; DSG2, desmoglein 2.

**Table 2:** Selected clinical studies with modified oncolytic viruses for HNC.

Virus Type	Virus Name	Gene Deletion/ Chimera	Transgene	Type of Tumors	Phase/Delivery	Dose of Virus	Ref.
Herpes simplex virus	T-vec	ICP34.5, ICP47,	GM-CSF	HNSCC	IT	10^6^–10^8^ PFU	[34]
	SKV-012	ICP34.5, ICP47	Survivin promoter-ICP34.5, IL-12	Solid tumor (HNSCC)	IT	10^6^, 10^8^ PFU/mL	[36]
	VT1093	ICP34.5, ICP47	Anti-PD-1	HNSCC	IT	1 × 10^7^–5 × 10^8^ PFU	[37]
	HF-10	UL43, UL49.5, UL55, UL56, LAT		Solid tumor (HMSCC)	IT	10^4^–10^6^ PFU	[38]
	T3011	ICP34.5	IL-12, Anti-PD-1	Solid tumor (HNSCC)	IT	10^8^ PFU/mL	[39]
	OH2	ICP34.5, ICP47	GM-CSF	Solid tumor (HNSCC)	IT	10^6^–10^8^ CCID50/mL	[40]
Adeno- viruses	ONYX-015, H101	E1B-55kDa		HNSCC	IT	2 × 10^11^ VP	[41]
	TILT-123 (Ad5/3- E2F-d24-hTNFα- IRES-hIL2)	24 bases in E1A, 5/3 chimera fiber	E2F promoter, TNFα, IL-2	Solid cancer (HNSCC)	IV IT	3 × 10^9^–4 × 10^12^ VP 3 × 10^9^–5 × 10^11^ VP	[42]
	Enadenotucirev (ColoAd1)	E2 chimera, E3, E4, 11p/3 chimera fiber		Epithelial cancer (HNSCC)	IV	1 × 10^12^–6 × 10^12^ VP	[43]
	NG-350A	E2 chimera, E3, E4, 11p/3 chimera fiber	Anti-CD40	Epithelial cancer (HNSCC)	IV IT	1 × 10^11^–6 × 10^12^ VP up to 1 × 10^12^ VP	[44]
	VCN-01	24 bases in E1A,	E2F promoter RGDK hyaluronidase	HNSCC	IV	3.3 × 10^12^–1 × 10^13^ VP	[7]
Vaccinia virus	GL-ONC1 (GLV-1h68, Olvi-Vec)	J2R(TK), F14.5L, A56R	RUC-GFP, β-galactosidase, β-glucuronidase	HNSCC	IV	3 × 10^8^–3 × 10^9^ PFU	[45]

HNC, head and neck cancer; HNSCC, head and neck squamous cell carcinoma; GM-CSF, granulocyte-macrophage colony-stimulating factor; IT, intratumoral; PFU, plaque-forming unit; LAT, latency-associated transcript; CCID, cell culture infectious dose; VP, viral particle; IV, intravenous; RGDK, Arg-Gly-Asp-Lys; RUC, Renilla luciferase; TK, thymidine kinase.

## Data Availability

No new data were created or analyzed in this study. Data sharing is not applicable to this article.
